# Institutionalization of evaluation in primary health care in Brazil: trajectory, advances, and contemporary challenges

**DOI:** 10.1590/0102-311XEN127126

**Published:** 2026-11-09

**Authors:** Luis Augusto Coelho Pisco

**Affiliations:** 1 Instituto Universitário de Lisboa, Lisboa, Portugal.

The authors ^1^ analyze the institutionalization of evaluation in primary health care (PHC) in Brazil, articulating a historical reconstruction of monitoring and evaluation policies with a theoretical reflection supported by contemporary references, among which transformative evaluation and planetary health stand out ^2^. Their proposal is relevant and timely, especially because it defends a concept of evaluation that goes beyond the strictly instrumental and managerial dimension, repositioning it as a strategic, ethical practice committed to the principles of the Brazilian Unified National Health System (SUS, acronym in Portuguese).

One of the most consistent aspects of the text ^1^ lies in its historical systematization of recent evaluation policies and instruments. Its retrieval of initiatives such as the Primary Health Care Information System (SIAB, acronym in Portuguese), the Primary Health Care Indicators Pact (PIAB, acronym in Portuguese), the Family Health Expansion and Consolidation Project (PROESF, acronym in Portuguese), the Improvement Access and Quality National Program of Primary Health Care (PMAQ-AB, acronym in Portuguese), and, more recently, Previne Brasil highlight the institutional and political displacements that shaped PHC evaluation. The text adequately shows that this process, rather than being restricted to the creation of normative instruments or information systems, articulates the Brazilian Ministry of Health, academia, and entities such as Brazilian Association of Public Health (ABRASCO, acronym in Portuguese) in a movement that combines technical production, political dispute, and the construction of institutional legitimacy.

Any reflection on the institutionalization of evaluation in primary health care must include the hybrid configuration of the Brazilian health system, in which a formally universal public sector coexists with an expanding private sector that exerts an increasing influence in the offers, flows, and social expectations around care. In such contexts, the question no longer revolves around how to better evaluate public services, including who is effectively subjected to evaluation devices under what objectives and with what degree of symmetry between the public and private sectors. By emphasizing State responsibility, the article focuses on the essentials. However, it could discuss whether the new evaluative architecture avoids the risk of reinforcing above all the unilateral accountability of public services, largely leaving private services to their own self-regulation.

Evaluation should be understood as part of a broader governance architecture that can reinforce transparency, improve policies, or serve as a formal and inconsequential control mechanism. The density of reflection on PHC increases when evaluation is linked to the democratic commitments by SUS and the need to produce public meanings for State action.

The article ^1^ pertinently mobilizes the framework of institutionalization, particularly based on Denis et al. ^3^ contributions; concepts such as legitimacy, sustainability, and institutional paradoxes are appropriately used to interpret the Brazilian case.

Evaluation must remain closely linked to the public purposes of SUS: universality, comprehensiveness, equity, and social participation. This is perhaps the strongest bet! It is not only rigor that legitimizes evaluation. The reconnection with the ethical and political purposes of the health system is essential lest evaluation runs the risk of remaining a language of intent without the ability to reorganize practices.

Another important merit of the article ^1^ is its expansion of health assessment by its incorporation of references associated with planetary health, sustainable development goals, resilience, and culturally responsive approaches ^4^.

The text explicitly defends SUS principles: universality, equity, and democratic governance. This choice is consistent with the critical tradition of Collective Health and gives ethical density to its argument. Moreover, the article avoids reducing evaluation to an exclusively technical instrument, acknowledging its inevitably political and debated nature.

Its approach to territory, resilience, and social transformations is one of the most innovative dimensions of the article ^1^. Its broadening of the understanding of territory beyond administration and incorporating themes such as violence, climate change, and job insecurity reinforces the need for evaluation processes that are more sensitive to social dynamics and structural inequalities.

To evaluate is to measure and to weigh the adequacy of policies, processes, and results regarding socially legitimate ends.

In a context in which performance-based management and financial incentives gain weight, evaluation can become less a means of institutional learning and more a mechanism for legitimizing prior decisions. Thus, a proposal for a more robust institutionalization should make explicit the importance of evaluation and the conditions that can protect it from short-term reasoning. The lack of this safeguard risks producing an evaluative apparatus that is formally sophisticated but politically untransformative.

Rather than an abstract space for standard application, PHC constitutes a relational field crossed by inequalities and concrete experiences of access, care, and medical attention continuity. An evaluation that intends to be meaningful must acknowledge this territorial dimension, articulating general indicators with the diversity of local contexts. Without such mediation, evaluations can become technically correct but socially insensitive.

Its final considerations adequately resume the main thesis of the article ^1^: the institutionalization of evaluation must go beyond the formalization of administrative instruments and routines, rooting itself in organizational practices, institutional relations, and public service ethics. This conclusion is consistent with the contemporary challenges of health management. Further strengthening this proposal could make explicit concrete implementation strategies and identify the institutional actors, governance mechanisms, and political conditions necessary for its viability.

Thus, the article offers a relevant contribution to the debate on evaluation in PHC in Brazil, articulating historical reconstruction, theoretical reflection, and normative positioning. Its main merits include its systematization of evaluation and monitoring policies, its analysis of the tensions inherent to the institutionalization process, and its incorporation of contemporary Collective Health references. Its limitations include the need for greater analytical depth of certain political-institutional contexts and the limited operationalization of its proposals. Still, the text fully fulfills its objective of fostering debate, offering important elements for more robust, legitimate evaluation policies in line with SUS principles.

The text offers an important contribution to rethinking evaluation in PHC, especially by rejecting it as a merely instrumental exercise. Its added value lies in placing evaluation back at the intersection between ethics, democracy, and management. It is necessary to further the institutional mediations that could convert this ambition into consolidated practice. The debate can move forward in the space between norm and implementation: from good principles to the conditions that make them operational.

## Data Availability

Not applicable.
