# Supplementary material for: Institutionalization of evaluation in primary health care in Brazil: trajectory, advances, and contemporary challenges
Source: Cad Saude Publica. 2026 Nov 9;42(Suppl 2):e00127126. doi: 10.1590/0102-311XEN127126 (PMC13750641; doi:10.1590/0102-311XEN127126)
Supplement: Translation

# Translation: Institucionalização da avaliação na atenção primária à saúde no Brasil: trajetória, avanços e desafios contemporâneos

**DOI:** 10.1590/0102-311XPT127126

**Authors:** Luis Augusto Coelho Pisco

**Affiliations:** 1 Instituto Universitário de Lisboa, Lisboa, Portugal.

Os autores ^1^ analisam o processo de institucionalização da avaliação na atenção primária à saúde (APS) no Brasil, articulando uma reconstrução histórica das políticas de monitorização e avaliação com uma reflexão teórica apoiada em referenciais contemporâneos, entre os quais se destacam a avaliação transformadora e a saúde planetária ^2^. A proposta é relevante e oportuna, sobretudo por defender um conceito de avaliação que ultrapassa a dimensão estritamente instrumental e gerencial, reposicionando-a como prática estratégica, ética e comprometida com os princípios do Sistema Único de Saúde (SUS).

Um dos aspetos mais consistentes do texto ^1^ reside na sistematização histórica das políticas e instrumentos de avaliação implementados nas últimas décadas. A recuperação de iniciativas como o Sistema de Informação da Atenção Básica (SIAB), o Pacto de Indicadores da Atenção Básica (PIAB), o Projeto de Expansão e Consolidação do Saúde da Família (PROESF), o Programa de Melhoria do Acesso e da Qualidade da Atenção Básica (PMAQ-AB) e, mais recentemente, o Previne Brasil permite evidenciar os deslocamentos institucionais e políticos que conformaram o campo da avaliação na APS. O texto mostra adequadamente que esse processo não se restringe à criação de instrumentos normativos ou sistemas de informação, envolvendo igualmente a atuação articulada do Ministério da Saúde, da academia e de entidades como a Associação Brasileira de Saúde Coletiva (ABRASCO), num movimento que combina produção técnica, disputa política e construção de legitimidade institucional.

Qualquer reflexão sobre a institucionalização da avaliação na APS não pode ser separada da configuração híbrida do sistema de saúde brasileiro, em que um setor público formalmente universal coexiste com um setor privado em expansão, cada vez mais influente na definição das ofertas, fluxos e expectativas sociais em torno do cuidado. Em contextos desse tipo, a questão deixa de ser apenas como avaliar melhor os serviços públicos e passa também a incluir quem é efetivamente submetido a dispositivos de avaliação, com que objetivos e com que grau de simetria entre público e privado. Ao enfatizar a responsabilidade do Estado, o artigo ^1^ incide no essencial, mas poderia discutir se a nova arquitetura avaliativa não corre o risco de reforçar sobretudo a responsabilização unilateral dos serviços públicos, deixando os privados, em grande medida, entregues à sua própria autorregulação.

A avaliação deve ser entendida como parte de uma arquitetura mais ampla de governação, na qual pode simultaneamente reforçar a transparência e o aperfeiçoamento das políticas ou servir de mecanismo de controle formal e pouco consequente. A densidade da reflexão sobre a APS aumenta quando a avaliação é ligada aos compromissos democráticos do SUS e à necessidade de produzir sentidos públicos para a ação do Estado.

O artigo ^1^ mobiliza de maneira pertinente o referencial da institucionalização, particularmente com base nas contribuições de Denis et al. ^3^. Conceitos como legitimidade, sustentabilidade e paradoxos institucionais são adequadamente utilizados para interpretar o caso brasileiro.

A avaliação deve permanecer estreitamente ligada às finalidades públicas do SUS: universalidade, integralidade, equidade e participação social. Essa é talvez a aposta mais forte! Não é, apenas, o rigor que legitima a avaliação. É fundamental reconectar-se às finalidades éticas e políticas do sistema de saúde. De outro modo, a avaliação corre o risco de permanecer como linguagem de intenção, sem capacidade de reorganizar práticas.

Outro mérito importante do artigo ^1^ consiste na ampliação do escopo da avaliação em saúde mediante a incorporação de referenciais associados à saúde planetária, aos Objetivos de Desenvolvimento Sustentável (ODS), à resiliência e às abordagens culturalmente responsivas ^4^.

O texto posiciona-se explicitamente em defesa dos princípios do SUS, da universalidade, da equidade e da governança democrática. Essa escolha é coerente com a tradição crítica da Saúde Coletiva e confere densidade ética ao argumento. Ao mesmo tempo, o artigo evita reduzir a avaliação a um instrumento exclusivamente técnico, reconhecendo o seu carácter inevitavelmente político e debatido.

A abordagem do território, da resiliência e das transformações sociais constitui uma das dimensões mais inovadoras do trabalho ^1^. Ao ampliar o entendimento de território para além da dimensão administrativa e incorporar temas como violência, mudanças climáticas e precarização do trabalho, o texto reforça a necessidade de processos avaliativos mais sensíveis às dinâmicas sociais e às desigualdades estruturais.

Avaliar não é apenas medir, mas ponderar a adequação de políticas, processos e resultados face a fins socialmente legitimados.

Num contexto em que a gestão por desempenho e os incentivos financeiros ganham peso, a avaliação pode tornar-se menos um meio de aprendizagem institucional e mais um mecanismo de legitimação de decisões já tomadas. Por isso, uma proposta de institucionalização mais concreta deveria explicitar não apenas a importância da avaliação, mas também as condições que permitem protegê-la da captura por lógicas de curto prazo. Sem essa salvaguarda, corre-se o risco de produzir um aparato avaliativo formalmente sofisticado, mas politicamente pouco transformador.

A APS não é um espaço abstrato de aplicação de normas; é um campo relacional, situado, atravessado por desigualdades e por experiências concretas de acesso, cuidado e continuidade assistencial. Uma avaliação que pretenda ser significativa precisa reconhecer essa dimensão territorial, articulando indicadores gerais com a diversidade dos contextos locais. Sem essa mediação, a avaliação pode tornar-se tecnicamente correta, mas socialmente insensível.

As considerações finais retomam adequadamente a principal tese do artigo ^1^: a institucionalização da avaliação não pode limitar-se à formalização de instrumentos e rotinas administrativas, devendo enraizar-se nas práticas organizacionais, nas relações institucionais e na ética do serviço público. Trata-se de uma conclusão consistente com os desafios contemporâneos da gestão em saúde. Para fortalecer ainda mais essa proposta, seria útil explicitar as estratégias concretas de implementação, bem como identificar atores institucionais, mecanismos de governança e condições políticas necessárias à sua viabilização.

Em síntese, o artigo oferece uma contribuição relevante para o debate sobre avaliação na APS no Brasil, articulando reconstrução histórica, reflexão teórica e posicionamento normativo. Entre os seus principais méritos destacam-se a sistematização das políticas de avaliação e monitorização, a análise das tensões inerentes ao processo de institucionalização e a incorporação de referenciais contemporâneos da Saúde Coletiva. Entre as limitações, sobressaem a necessidade de maior aprofundamento analítico de determinados contextos político-institucionais e a limitada operacionalização das propostas apresentadas. Ainda assim, o texto cumpre plenamente o objetivo de fomentar o debate e oferece elementos importantes para a construção de políticas de avaliação mais concretas, legítimas e alinhadas aos princípios do SUS.

O texto oferece uma contribuição importante para repensar a avaliação na APS, sobretudo ao recusá-la como exercício meramente instrumental. A sua mais valia está em recolocar a avaliação no cruzamento entre ética, democracia e gestão. É necessário aprofundar as mediações institucionais que permitiriam converter essa ambição em prática consolidada. É no espaço entre norma e implementação que o debate pode avançar: passar dos bons princípios às condições que os tornem operativos.

## Data Availability

Não se aplica.
